# Lung Cancer Europe challenges stigma: an interview with Anne-Marie Baird

**DOI:** 10.1242/dmm.050606

**Published:** 2023-12-20

**Authors:** Anne-Marie Baird

**Affiliations:** ^1^ School of Medicine, Trinity Translational Medicine Institute, Trinity College Dublin, D08 W9RT, Ireland; ^2^ Lung Cancer Europe, Effingerstrasse 40, 3008 Bern, Switzerland

Lung cancer is the leading cause of cancer death in European Union countries, as it was responsible for approximately one out of five cancer-related deaths in 2020 according to the European Commission. This is primarily due to late diagnosis often caused by delayed onset of symptoms in late stages of cancer. There are two types of primary lung cancer – non-small cell and small cell lung cancer – with non-small cell lung cancer being the more common, but slower spreading, type. Lung cancer is most frequently associated with chronic cigarette smoke inhalation, but roughly 10–20% of cases are diagnosed in those with no history of smoking (Cufari et al., 2017; Siegel et al., 2021). Other risk factors exist, including exposure to other carcinogens, such as radon, and genetic predisposition. Improvements in diagnostics and the wide implementation of screening are needed to detect lung cancer at earlier stages when treatments are usually more successful. Furthermore, in recent years a deeper understanding of the molecular changes that drive lung cancer growth has led to the introduction of targeted therapies and immunotherapies.

Dr Anne-Marie Baird is the President of Lung Cancer Europe (LuCE; Box 1) and is also a Senior Research Fellow at Trinity College Dublin, Ireland. Her research focuses on inflammation, metastasis, drug resistance and biomarkers in lung cancer. In this interview she discusses her personal motivations to work in patient advocacy and how her research background helps connect people with lung cancer with scientists to ensure research is prioritizing patient needs.

**Figure:**
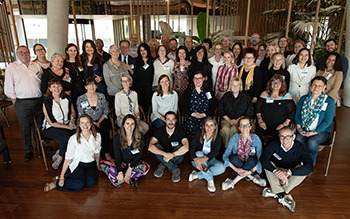
**LuCE AGM Amsterdam, May 2023.** This image is courtesy of Lung Cancer Europe and published under the CC-BY 4.0 license for this article.

**Box 1. Lung Cancer Europe**

Lung Cancer Europe (LuCE) is a non-profit organisation spanning 24 European countries that connects and supports people living with lung cancer and their families. One of the key aims of LuCE is to increase access to early and accurate lung cancer diagnosis across Europe. This involves awareness campaigns, like ‘Get Checked!’ that educates the public about the risk factors and symptoms associated with lung cancer, and highlights that ‘Anyone with lungs can get lung cancer’. This campaign also acts to destigmatize the disease by looking beyond the association with smoking. LuCE also aims to improve access to cutting-edge treatments and care for all people impacted by lung cancer. By composing in-depth reports and position papers, they are informing policy makers and other stakeholders in the community to take action to improve lung cancer diagnosis and treatment across Europe (https://www.lungcancereurope.eu/).

## Could you tell us about your career, and when and why you decided to become a patient advocate?

I was always interested in science, so when I went to university, I chose to study the biological sciences. My specific interest in cancer research stemmed from the loss of my grandfather from bowel cancer when I was 16. I did a PhD and a few postdocs, all within the lung cancer space. During my PhD, my aunt was diagnosed with small cell lung cancer, and this was the second lung cancer diagnosis in my family, as my grandmother had also died from the disease. This felt sort of strange, as I was in the lab, doing experiments on lung cancer cell lines, but I never really thought of ‘the other side’ – the people experiencing the disease. Pretty much immediately after her diagnosis, I experienced the stigma associated with lung cancer. People would say, “Did she smoke? Well then, what did you expect?” and there was very little empathy. This experience drove me into patient advocacy. Although I guess I didn't always stand my ground – I'm a bit too Irish sometimes – so at that time, I was afraid to get too involved. It took a while to find my voice and my courage. So, it was only in 2012 that I became formally involved in lung cancer patient advocacy. First through social media, especially Twitter, then things grew organically from there. I'm now in a very privileged position where I have both my research career at Trinity College Dublin with a really supportive PI and can carry on with my advocacy work.

Our advocacy community uses the phrase “Anyone with lungs can get lung cancer” in our messaging, and I think this is something the public needs to be aware of.

## Could you tell us about the prevailing lung cancer stigma?

Lung cancer stigma is very strong, and we could talk about it all day. It affects people every day, even leading some to lie about their diagnosis to avoid judgement. A cancer diagnosis is devastating enough, but having to face prejudice or feeling the need to hide it from loved ones adds a significant burden. And the effects are not just on the psychological level, they are much broader than that. Some of the public do not see lung cancer as ‘blue’ enough or ‘pink’ enough, and there is a lack of awareness that lung cancer kills more people than prostate and breast cancers combined. The general public is aware of the typical symptoms of breast cancer but this is generally not the case for lung cancer. Additionally, because of the persistence of the link to smoking, most people who never smoked believe they are not at risk for lung cancer, which – again – is not true. And this bias can also persist in primary care where there may be a failure to consider a lung cancer diagnosis in those without a smoking history. Our advocacy community uses the phrase “Anyone with lungs can get lung cancer” in our messaging, and I think this is something the public needs to be aware of.

## In your advocacy work, what is your main goal?

As an advocate, my goal is to raise the voice of people impacted by lung cancer. For a really long time, patients were not asked about their priorities. And previously many regarded lung cancer itself as an uninteresting area of research. This of course changed with the advent of molecular diagnostics and oncogene-targeted therapies. We now know a lot more about the biological complexity of the disease, so it has become more attractive for further research, which translates into more treatments becoming available in the clinic. So, my goal as a lung cancer researcher and patient advocate is to ensure the patient voice is part of the process. Obviously, I do not have lung cancer myself, so I am very conscious when talking to others and advocating for people who live with this disease every day. Their needs, wants and priorities need to be accurately translated both to the clinical side and the research side. In a way, I try to act as the bridge between these communities. We are currently not at the point where these are integrated, so trying to be this bridge is where I see one of my purposes in patient advocacy.

## Because you live both worlds – lung cancer research and lung cancer advocacy – can you tell us how incorporating advocacy views into research can benefit both people with lung cancer and the research itself?

People with the disease, are the ones who undergo diagnostic procedures and go through all the treatments. And they experience all the ups and downs associated with all of this, as do their family and friends. They are the ones who can tell us what works, what doesn't work and what's still missing. This spans diagnostics, treatments, side effects, supportive therapies, etc. This gives researchers an understanding of the real-world priorities. Sometimes this can mean going back to basics, switching the research question to balance treatment efficacy with quality of life and so on. Researchers can sometimes lose sight of that when we get wrapped up in things like grants and publications, but we should aim for a research culture where we can speak to the people with the disease and ask them what it is that they value most, and form our research questions from there. This way, our work in the lab really has the potential to make a difference. This may be an overly simplistic view but it is how, I think, we should be working, humanizing our research. We can sometimes develop tunnel vision in the lab, as we are very focused on finding the answer to our very specific grant objective. But we need to be mindful of what the long-term goal of all this research is.

I know this can be a challenge to some researchers. Many of us may think “What if people ask for things I can't deliver and I let them down?” or “What if they won't be able to understand the science?”. Often, it's also an issue of time, and there may be a worry that engaging with people impacted by cancer may delay work or their schedule may not be compatible with that of the researchers. But funders are placing more emphasis on PPIE, which is public and patient involvement and engagement, so researchers need to engage. Avoiding the conversation before it even starts is not an option anymore. Researchers try to educate the public, and people living with the disease should have the opportunity to educate researchers and the public, too.

## So this means people with lung cancer should be empowered to speak to researchers?

I think this is essential. Lung cancer research is still severely underfunded compared to other cancers, so setting the right research priorities is essential to maximize progress within this constrained funding. And as we discussed earlier, PPIE is essential in setting research and funding priorities. However, one of the barriers for many people living with lung cancer is the perceived and, often, real feeling that their input will not be valued, and it will amount to a ‘box-ticking’ exercise to fulfil a requirement. People who actively get involved must be valued and the door should be kept open for more to join the conversation when they feel comfortable to do so.

## As an advocate, what would you most like to tell researchers?

I'd first tell them not to be scared of reaching out to advocates. Many researchers may dismiss their outreach because they believe either nobody will understand or care about the science. As a first step, a researcher can establish contact with their local patient support group(s) and offer a meeting, presentation or similar. The truth is, not every advocate or patient group will respond immediately, but making the effort to reach out will help to establish that first contact and start the conversation. This must be a two-way street and will need effort from both sides, and will require active engagement. At the same time, I'd recommend approaching outreach as a meaningful and valuable part of research priority setting.

## When talking to members of your organisation, what are the main concerns they voice?

In the lung cancer space, the main concern is equitable access to diagnostics, treatments and care. At the moment, where a person lives and their socioeconomic status dramatically affects their access to care. We see this in disparities in survival; and this applies to several cancers, not just those of the lung. First, for lung cancer in particular, screening is a particular problem. Very few countries in Europe have developed a lung cancer screening programme, despite data showing that such programmes are effective (de Koning et al., 2020). So, this is something that is not available for the vast majority of people who are at risk of developing this disease. Second, access to proper diagnostics. A word I often hear our members say is ‘lucky’. Some say, “I was very lucky my consultant sent my sample for molecular testing”. You don't hear this word as often in other cancers, where molecular subtyping is part of established standards of care. It can't be down to luck! In the era of molecular diagnostics, people need an accurate diagnosis of their disease as it can make a huge difference to treatment plans and outcomes. And third, there still is a disparity between scientific discovery and clinical translation. People read about all the amazing advances, but how amazing are those if they do not (yet) translate to diagnostic and treatment progress in the real world. We need a way to translate these so every person can benefit.

## Can you think of any examples where advocates have helped set research priorities for lung cancer?

There's significant variation. In some countries, like the US, lung cancer patient advocacy has a much longer history, whereas in many European countries it is still somewhat new. One of the reasons for this variation is language. Although it's easy to assume that most EU citizens have a working knowledge of English, this is not always the case. And it means that a lot of resources and information coming from English speaking countries are not accessible, and it can also mean that it's difficult for advocates to find a common unifying voice.

There are now many specific oncogene-driven patient groups, two examples of which are the EGFR Resisters and ROS1ders, who have significantly raised awareness about their disease and funds for research. The ROS1ders have spearheaded the development of ROS1-mutant cancer cell line models, a crucial tool that was not available before. EGFR Resisters have also driven a lot of research, not just at the bench, but also studies on quality of life, treatment side effects and other aspects relevant to those living with EGFR-mutant lung cancer. This shows the significant impact of patient-driven research.

There is no real benefit to the community as a whole if only a selected few have access to advanced therapies. Anyone who could benefit from innovative treatment, must have access to it.

## Where do you think the field will be in the next five years?

I'd like to see screening programmes become available across Europe. This should really help with the ‘stage shift’, where more people are diagnosed at an earlier stage. I also hope for more investment in research so we can find out more about the biology of lung cancers and develop more-effective therapies. We need to aim for therapies that not only extend life but provide adequate quality of life with a reduced side effect profile. And even when those drugs are available, we need to work towards ensuring they are accessible to all, regardless of their country of residence or socioeconomic status. There is no real benefit to the community as a whole if only a selected few have access to advanced therapies. Anyone who could benefit from innovative treatment, must have access to it.

## We discussed the importance of advocacy in relation to research, but what is its value in policymaking and legislation?

The link between lung cancer and smoking is often a barrier for politicians. Advocacy can make a tremendous difference in breaking the stigma associated with lung cancer. We have already started to chip away at it, but we're still some way away from the paradigm shift; we need to have lung cancer as a healthcare policy priority.

## Conclusions

After a diagnosis with lung cancer, only two in five people can expect to reach one year of survival according to the National Health Service, UK. Organisations like Lung Cancer Europe are striving to change this through initiatives to improve diagnosis and treatments. The prevailing stigma surrounding lung cancer not only has a psychological effect but can have serious consequences in terms of diagnosis and funding. Greater awareness, alongside screening programmes, is required to combat these challenges and encourage earlier diagnosis that generally leads to better treatment outcomes. Furthermore, advances in molecular diagnostics and targeted therapies need to be accessible to all. The work done by Anne-Marie and her colleagues at Lung Cancer Europe is making tangible improvements in this space. They are determined to destigmatize the disease and ensure everyone impacted by lung cancer can benefit from clinical advances.
